# Emergent data influences the risk/benefit assessment of hemophilia gene therapy using recombinant adeno-associated virus

**DOI:** 10.3389/fmed.2023.1256919

**Published:** 2023-11-10

**Authors:** John Puetz

**Affiliations:** Department of Pediatrics, Division of Pediatric Hematology/Oncology, Saint Louis University School of Medicine, St. Louis, MO, United States

**Keywords:** hemophilia, gene therapy, adeno-associated virus, factor VIII, factor IX, hepatocellular carcinoma, thrombotic microangiopathy

## Abstract

After decades of investigation, gene therapy has received regulatory approval to treat hemophilia. However, since gene therapy investigations were initially conceived, other avenues of treatment have revolutionized the care of hemophilia. Emergent data is showing that gene therapy may not be as beneficial as hoped and more toxic than planned. At a minimum, a reassessment of risk/benefit estimate of gene therapy for hemophilia is needed.

## Introduction

Over the past few years, multiple human gene therapy trials have successfully achieved hemostatic levels of factors VIII and IX using recombinant adeno-associated virus (rAAV) vectors (1, 2). As a culmination of decades of work, rAAV vectors have received regulatory approval for treatment of both hemophilia A and B. Unfortunately, over the past few years, data has emerged that now calls into question the wisdom of using rAAV vectors to treat hemophilia A and B. This manuscript will review current treatments of hemophilia, benefits of gene therapy, and the risks of gene therapy. Although other vectors are in development, since rAAV is the principal vector used for gene therapy in hemophilia, this manuscript will focus on rAAV toxicity. At a minimum, a reassessment of the risk/benefit estimate before routinely administering rAAV vectors to treat hemophilia seems prudent.

## Current treatment of hemophilia

Hemophilia A and B are bleeding disorders in which patients bleed abnormally from trivial causes. Prior to modern treatment, patients suffered from recurrent abnormal bleeding leading to crippling, painful, arthropathies and premature death. Starting in the 1960’s and 70’s, factor replacement became available and was given in the home (3, 4). Bleeding episodes could finally be controlled. Patients underwent elective surgeries. However, joint disease and other complications persisted. Life expectancy improved but remained far from normal. Factor replacement led to alloimmunization (inhibitor) in some patients, which complicated treatment immensely. In the 1980s contamination of factor concentrates with HIV and hepatitis C devastated the hemophilia community (3, 4). In this environment, alternatives to episodic plasma derived factor replacement, including gene therapy, were proposed (5). Fortunately, advances in treatments in the 1990s started to turn the tide on the complications of hemophilia. Recombinant factor products were developed and improvements in plasma derived products eliminated the risk of HIV and hepatitis C transmission (4). Prophylactic infusion of factor reduced joint bleeding and other complications (6). Life expectancy improved further. However, the burden of treatment was significant. Prophylactic regimens required intravenous infusions at least twice weekly, and daily to achieve immune tolerance for those alloimmunized (6, 7). To facilitate the infusion, indwelling catheters were used. This led to infectious and thrombotic complications (8). The 2010s saw further improvements in treatment. Extended half-life products were developed that reduced the burden of prophylactic treatment (9, 10). Patients with hemophilia A and B are now treated with prophylactic regimens with once weekly (or less) infusions (9, 11). The factor VIII mimetic Emicizumab has revolutionized the treatment for hemophilia A (12–14). The annualized bleeding rate (ABR) for children with hemophilia A (alloimmunized or not) is approaching zero with subcutaneous injections of Emicizumab (13). This can be given as infrequently as once monthly (13, 14). The life expectancy of people with hemophilia is now approaching normal (15). Additional improvements in treatments are on the horizon. Treatments aimed at rebalancing hemostasis are nearing regulatory approval (14, 16, 17). Thus, a subcutaneous treatment for hemophilia B (alloimmunized or not) may soon be available. In diseases other than hemophilia, extended half-life strategies for monoclonal antibodies have been developed with therapeutic levels lasting 6 months (18). Similar technologies could be applied to hemophilia treatments, reducing the need for subcutaneous injections to 2–3 times a year.

## The hope of gene therapy

While the life expectancy of people with hemophilia is near normal, it is not normal (15). Joint bleeding has been reduced, but not eliminated (6, 9–14). ABRs are approaching zero, but are not zero (9–14, 16, 17). The burden of treatment has been drastically reduced but persists. Treatments are not accessible to large populations of patients and where they are, patients do not always adhere to recommended treatments (19). The risk of transmitting infectious agents or other manufacturing mishaps remains (20). Emicizumab may increase the risk of thrombotic events (21). The financial costs of treatment remain substantial. In short, there remains room for improvement in the treatment of hemophilia. Current gene therapy regimens may fulfill that need. A one-time treatment with rAAV vectors has resulted in hemostatic levels for both factor VIII and IX (1, 2). Long term data is still needed, but current rAAV gene therapy may further reduce or eliminate joint disease and other bleeding complications. Although the financial cost of gene therapy is substantial, if hemostatic levels are sustained, the lifetime costs of treatment will be reduced (22). The most significant side effect of rAAV gene therapy for hemophilia during clinical trials has been mild liver inflammation, which has generally been controlled with steroids (1, 2). Although the specter of genotoxicity has been raised, none has been observed to date in human trials of rAAV for hemophilia. Thus, it has been claimed that it is time to “prepare the way” for hemophilia gene therapy (23). Or is it?

## The reality of gene therapy

While the hope of gene therapy is curative treatment, the reality is otherwise. As shown in Table 1, currently approved treatments are not curative (9, 11, 12, 24, 25). Even if not curative, a reduced burden of medication administration and a “bleed-free” mindset would still be beneficial (26). Although the clinical manifestations of hemophilia A and B are similar, they really are two different diseases. This is evident from the results of the phase 3 hemophilia gene therapy trials (24, 25). In both trials, hemostatic levels were achieved, however, there were substantial differences in durability and toxicity.

**Table 1:** Compares curative treatment, current gene therapy, and standard of care.

		Hemophilia A			Hemophilia B	
Feature	Curative treatment	Valoctocogene roxaparvovec	Emicizumab	EHL FVIII	Etranacogene dezaparvovec	EHL FIX
One-time treatment	Yes	Yes	No	No	Yes	No
Given prenatally or early neonate	Yes	No	Yes	Yes	No	Yes
Option for all patients	Yes	No	Yes^*^	No	No	No
Normal factor activity following treatment	Yes	No	No	Possible	No	Possible
Hemostatic factor activity for all treated patients	Yes	No	Yes^$^	Yes^$^	No	Yes^$^
Predictable response	Yes	No	Yes	Yes	No	Yes
Sustained for a lifetime (80+ years)	Yes	No	Yes	Yes	No	Yes
Mean annualized bleeding rate	0	1.2	2.5	0.7	1.5	1.6
Eliminate need for factor replacement for all patients in all situations	Yes	No	No	No	No	No
Risk for unintended genome insertion	No	Yes	No	No	Yes	No
Risk for malignancy	No	Unknown	No	No	Unknown	No
Risk for hepatic inflammation	No	Yes	No	No	Yes	No
Risk for hepatic failure with vector^&^	No	Yes	No	No	Yes	No
Risk for TMA with vector^&^	No	Yes	Yes^#^	No	Yes	No
Risk for DRG toxicity with vector^&^	No	Yes	No	No	Yes	No
Risk for other toxicity with vector^&^	No	Yes	No	No	Yes	No
Risk of reduced bone density	No	Unknown	Unknown	Unknown	Unknown	Unknown

EHL-enhanced half-life; TMA-thrombotic microangiopathy; DRG-dorsal root ganglion. ^*^Access may be limited in various parts of the world, but it is available and is approved for use in patients with mild, moderate, and severe hemophilia with or without an inhibitor. ^$^Other than patients who become alloimmunized. ^&^Not reported in clinical trials for hemophilia or spinal muscular atrophy (SMA) but has been reported in post-marketing surveillance for SMA and in clinical trials for other genetic disorders. ^#^Can be avoided by limiting co-exposure to bypass agents containing factor IX.

In the hemophilia B study, 52 of the 54 subjects were able to achieve a mean factor IX activity above baseline of 36 IU/dL at 6 months and dropped minimally by 18 months to 34 IU/dL above baseline (25). Based on this, it has been estimated that >80% of subjects will maintain factor IX level > 2% at 25 years post treatment (27). The need for factor replacement was reduced when compared to the lead-in period. The ABR was also reduced when compared to the lead-in period. However, only 57% of the subjects were on an extended half-life product during the lead-in period. As shown in Table 1, the ABR following gene therapy showed no ABR reduction when compared to factor IX extended half-life studies (9, 25). The principal toxicity reported was elevation in liver transaminases. This occurred in 20% of subjects and was controlled with a course of steroids (median 11 weeks). The gene construct used for the trials was the gain of function factor IX Padua variant. This variant has never been used for human factor IX replacement prior to the gene therapy trials. Thus, the risk of inhibitor development is unknown. Extravascular factor IX binding to collagen type IV is also important for hemostatic activity (28). Also unknown is the factor IX Padua variant extravascular hemostatic activity in humans with hemophilia B.

For the hemophilia A trial, subjects achieved a median factor VIII activity of ~55 IU/dL at 6 months (24). Unfortunately, this was not durable. Within 2 years, roughly 5% of subjects had returned to prophylaxis. Within 5 years, factor VIII activity is estimated to drop to 6 IU/dL. Factor usage and the ABR fell compared to the lead-in period. However, none of the subjects were treated with Emicizumab or Efanesoctocog during the lead-in period (24). As shown in Table 1, the ABR following gene therapy was marginally better compared to clinical trial results of Emicizumab, and higher than trial results of Efanesoctocog (11, 12, 24). Again, the principal toxicity was elevation in liver transaminases. However, this was much more prevalent and recalcitrant to treatment compared to the hemophilia B study. Nearly 90% of hemophilia A subjects developed elevated transaminases. This was mostly treated with glucocorticoids alone for a median of 33 weeks. Roughly 20% of subjects received immunosuppression beyond glucocorticoids including tacrolimus and mycophenolate. The gene construct use was a b-domain deleted factor VIII, This variant has been used extensively for human factor VIII replacement. The dose of vector used during the hemophilia A trial was 3 times higher than the hemophilia B trial (6 × 10^13^ vg/kg vs. 2 ×10^13^ vg/kg) (24, 25). It is unknown to what extent this dosing difference may have contributed to the difference in toxicity.

Common to both studies was wide variability in response. Some subjects did not respond at all while others achieved supratherapeutic levels. For those subjects who did not respond or had an unsustained response, redosing with the same construct is not feasible. Potentially, patients can receive subsequent treatment with different vectors and constructs, but this needs to be investigated. Neither phase 3 study described treatment strategies used for surgical procedures. Based on the mean factor levels achieved, it is anticipated that a sizable proportion of subjects will still need factor replacement for surgical procedures.

In summary, the principal benefit of AAV gene therapy seen thus far is not a dramatic reduction in bleeding risk, but the need for frequent medication administration. Thus, gene therapy provides an added measure of convenience and potentially reduced cost. For hemophilia B, this benefit may outweigh the known risks described in the clinical trials. For hemophilia A, this is less clear.

However, there may be additional risk to hemophilia gene therapy with AAV vectors that were not evident in the clinical trials.

## Additional risks of gene therapy

### Hepatocellular carcinoma

The Adeno-associated virus was discovered in the 1960s and for decades was not thought to cause any human disease (29). For this reason, it was an ideal candidate for use as a gene therapy vector. However, Nault et al. reported wild type AAV (wAAV) insertions near the *TERT* promoter in human hepatocellular carcinoma (HCC) specimens (30). Additional wAAV insertions near 4 other oncogenes were found, and the insertions were clonal (30). These same oncogenes are targeted by hepatitis B, which is known to contribute to HCC (31). There remains debate as to whether the wAAV insertions are drivers of oncogenes or benign passengers (32). As far as hemophilia gene therapy goes, it really does not matter if wAAV causes HCC or not. wAAV is not infused into patients for gene therapy, rAAV is. The question for hemophilia gene therapy is does rAAV cause HCC?

Unfortunately, the answer is yes, and here there is no debate (33). Pre-clinical Investigations of rAAV gene therapy date back over 2 decades. An early investigation of rAAV involved a mouse model of mucopolysaccharidosis VII (MPSVII). Neonatal mice with this disorder were successfully treated with rAAV. However, a small number developed HCC (34, 35). Because MPSVII is fatal in mice, it was unclear if the rAAV was causing the HCC or the underlying disease was. A follow-up study included a normal control group. These neonatal mice also developed HCC following rAAV (34). Investigation over the ensuing 2 decades showed that rAAV inserts and drives the *RIAN* locus, more specifically microRNA-341, in neonatal mice. This does not occur in adult mice. Humans do not have a *RIAN* ortholog, but dogs do (35). The combination of rAAV/neonatal mouse/*RIAN* locus are needed to induce HCC, or so it was thought (34).

Numerous trials of rAAV gene therapy for a variety of genetic disorders have been carried out in adult mice without the development of HCC (36). One difference in neonatal and adult mice is that neonatal mice have more proliferating cells, notably, hepatocytes. A recent investigation of rAAV gene therapy tested if rAAV could induce HCC in adult mice with liver injury (proliferating hepatocytes) (36). Investigators used either partial hepatectomy or fatty liver disease (high fat diet). Two rAAV vectors were used, one designed to drive the RIAN locus, and another vector control with a reporter gene. As expected, neither vector caused HCC above baseline in control adult mice. The rAAV vector designed to drive the RIAN locus caused HCC in both models of proliferating hepatocytes. Surprisingly, so did the control vector in adult mice with fatty liver disease (partial hepatectomy not tested) (36). In addition, a clinical trial of rAAV for phenylketonuria was placed on hold due to the development of HCC in a mouse model (2). Thus, rAAV can induce HCC in adult mice with proliferating hepatocytes. What about other animal models?

To date, HCC has not been observed in dogs or non-human primate rAAV gene therapy for hemophilia. However, clonally proliferating liver cells have been found (37). Nguyen et al. recently reported on a long term follow up of hemophilia A gene therapy in dogs using rAAV (37). Nine dogs were followed for up to 10 years. Liver samples were available in 6 of the dogs. rAAV integrations were found in 1,741 sites, and between 1 and 130 cells per integration. Preferential integrations near oncogenes in clonally proliferating cells were seen. The dogs were otherwise healthy. The authors did not report on the presence or absence of fatty liver disease. A second long term study of 8 hemophilic A dogs receiving rAAV gene therapy did not find clonal proliferation in the liver (38). This manuscript did not report on rAAV integrations, and additional analysis may be forthcoming. Long term data for hemophilia B gene therapy in dogs is also available (39, 40). Many canine gene therapy strategies for hemophilia B using rAAV targeted skeletal muscle and may not confer the same HCC risk as strategies targeting hepatocytes. There are no reports of HCC. However, detailed analysis for rAAV insertions and clonal proliferation have not been presented.

One subject in a human trial of rAAV for hemophilia B developed HCC (35). This subject had risk factors for developing HCC including hepatitis B and C. His liver specimen has been extensively evaluated and did not reveal genetic changes related to rAAV carcinogenesis but did have genetic changes typically found in HCC (35). No other human subjects have been reported with HCC following gene therapy for hemophilia. A recent report of liver biopsy results from 5 subjects who participated in a hemophilia A gene therapy trial did not show evidence of HCC or clonal proliferation (41). Notably, 4 of 5 subjects had liver steatosis, which apparently was subclinical.

In summary, emergent data has shown that rAAV can induce HCC in adult mice with liver disease (36). Long term studies of small number (<15) of hemophilia A dogs have shown clonal proliferation in the liver (37, 38). If rAAV causes HCC in a small proportion, or even 5%–10% of hemophilia A dogs, it could easily be missed by the current canine studies. To date, no studies have looked at the risk of HCC development of dogs or non-human primates with fatty liver disease following hemophilia AAV gene therapy. No humans have developed HCC or clonal proliferation caused by rAAV. Since the latency for the development of human HCC following rAAV may be decades, the risk of human HCC following rAAV cannot currently be estimated.

### Genome integration

Another advantage of using rAAV as a vector is that it mostly remains episomal following insertion into a cell (42). Until recently, studies have suggested that 99^+^% of rAAV vector was episomal, and <1% integrates. Emergent data has shown a higher percentage of integrations. Up to 3% of rAAV may integrate into liver cells following gene therapy (42). Most hemophilia gene therapy protocols infuse 10^14^–10^15^ viral particles and target 10^11^ hepatocytes. Assuming a more conservative estimate of genome integrations of 0.1%, one could anticipate over 100 million integrations following gene therapy. Indeed, some experimental data confirms this notion (42). Therefore, there are a massive number of integrations following rAAV gene therapy. Most are not intact vectors. With 10^11^ hepatocytes in a human adult, each with a genome of 3 × 10^9^ base pairs, most integrations are likely to land in an inactive genetic region if they integrate randomly. However, the above referenced dog hemophilia gene therapy study suggests that integrations are not random. They tend to occur near active genes, including oncogenes (37). One driver integration near the wrong oncogene at the wrong time could start the cell toward clonal proliferation and eventually, over years or decades, overt cancer.

### Other cancers

In addition to the above-mentioned HCC following rAAV gene therapy for hemophilia, 3 additional cases of cancer have been reported in humans, and one in a dog (43–45). As with the HCC, the other cancers (tonsillar carcinoma, salivary gland carcinoma, leukemia) have been thoroughly investigated and shown not to be caused by rAAV (43–45). It is unknown if immunosuppression received during clinical trials may have contributed to oncogenesis. To date, several hundred subjects have participated in clinical trials involving rAAV for hemophilia with reported observation periods lasting several years. There is easily over 1,000 person years of observation. Thus 4 reports of human cancer may not be unexpected. However, a proper epidemiological investigation seems indicated.

### Unfolded protein response

The target cell for hemophilia gene therapy using rAAV is the hepatocyte. Although factor VIII is made in the liver, it is not made in the hepatocyte (46). Accordingly, hemophilia A gene therapy targets a cell that does not typically produce factor VIII. Factor IX is naturally made in the hepatocyte. Factor VIII is a large protein with complex folding. Misfolded factor VIII protein can lead to cellular toxicity via the unfolded protein response (UPR) (47). This has been shown to occur when non-native cells are driven to express factor VIII. This occurs both *in vitro* (Chinese hamster ovary cells) and *in vivo* (mouse hepatocytes) for factor VIII (47, 48). Factor IX expression is stable for years following rAAV gene therapy in animal models and humans (1, 2). While factor VIII expression has been stable following rAAV gene therapy in animals, this has not been the case in humans when therapeutic levels are achieved. Several clinical trials of AAV gene therapy for hemophilia A have shown that hemostatic levels are not sustained (24, 49). The etiology for the falling levels remains unclear. An immune response has been investigated and does not clearly seem to be the cause (35). UPR could provide another explanation. However, as above, liver biopsies from human subjects following hemophilia A gene therapy failed to demonstrate evidence for UPR at the time of biopsies (41). Biopsies were performed 2.6–4.1 years following infusion of rAAV. So cellular toxicity/loss from UPR occurring prior to this would have been missed.

Although an immune response and UPR have been independently proposed as explanations for falling factor VIII expression following AAV gene therapy, an investigation by Butterfield et al. suggests that they may not be mutually exclusive (50). In addition, this study also suggested that translational shutdown (related to UPR and immune response) rather than loss of hepatocytes could lead to falling factor VIII expression following AAV gene therapy.

Concerns about UPR and HCC have also been raised. Kapelanski-Lamoureux et al. have investigated UPR and HCC risk in mice (51). In their study, all mice fed a high fat diet following receipt of a B-domain deleted factor VIII gene therapy vector (non-AAV) via hydrodynamic tail vein injection developed liver tumors. This happened less so with a factor VIII variant vector less prone to misfolding and not at all with a control vector. This suggests that factor VIII misfolding in mice fed a high fight diet contributes to the development of HCC independent of viral vector integration.

### Spinal muscular atrophy

One of the first rAAV gene therapy treatments to achieve regulatory approval was for the treatment of spinal muscular atrophy (SMA). This is a degenerative neuromuscular disorder that results in early death in those affected with severe (infantile) forms. rAAV gene therapy for this disorder has met with widespread success (52). While affected infants treated with rAAV gene therapy are not normal, they are achieving developmental milestones with an extended lifespan. Like hemophilia rAAV trials, the principal toxicity seen during the SMA trials was mild liver inflammation that is controlled with steroids (53). Now that over 3,000 infants have received gene therapy for this disorder, rare side effects not seen during the clinical trials are being observed (52). At least 9 cases of thrombotic microangiography (TMA) have been reported in the medical literature following rAAV gene therapy for SMA, one of which was fatal (54). Thirty (two fatalities) cases of TMA are reported in the U.S. (United States) Food and Drug Administration Adverse Event Reporting System (FAERS) (55). It is unknown if there is any overlap between cases reported in FAERS and the medical literature. The manufacturer of SMA rAAV has reported two cases of hepatotoxicity leading to fatalities (56). FAERS reports 120 cases of hepatobiliary disorders and 8 fatalities (55). One should interpret the FAERS data with caution, as the cause of death is not listed, only “Reactions.” Duplicate reports may also be present. Therefore, patients who died from causes unrelated to rAAV may be included in this database. Table 2 shows the number of reported relevant “Reactions” and deaths in the FAERS database for regulatory approved medications for SMA (55–59). A comparison of clinical trial results suggests that the event free survival rate is similar between Onasemnogene abeparvovec and Risdiplam, and higher than Nusinersen (59). Post-marketing surveillance from the FAERS database also shows higher mortality reporting for Nusinersen. TMA and hepatobiliary disease following treatment for SMA seems to be relatively unique to Onasemnogene abeparvovec. Use of rAAV in other clinical trials has also resulted in hepatotoxicity related fatalities (54). Because SMA and hemophilia are different diseases, different age groups were treated, and different doses used, similar toxicities may not occur in hemophilia patients following regulatory approval for rAAV gene therapy. However, the potential for rare, serious, and potentially fatal toxicities should be included in any risk/benefit calculation.

**Table 2:** Reactions and deaths reported in the Food and Drug Administration Adverse Event Reporting System for medications approved to treat spinal muscular atrophy.

	Number treated	Deaths	TMA	Hepatobiliary disorders
Onasemnogene abeparvovec (rAAV)	3,000	72	30	120
Risdiplam (RNA splicing modifier)	5,000	80	0	9
Nusinersen (anti-sense Oligonucleotide)	13,000	536	2	31

TMA, thrombotic microangiopathy; rAAV, recombinant adeno-associated virus.

### Dorsal root ganglion

Dorsal root ganglion (DRG) pathology has been commonly found in non-human primate gene therapy using rAAV (60). This has been found in a variety of vectors for a variety of diseases. It occurs more commonly with central nervous system administration but is also seen following intravenous administration of rAAV. Immunosuppression does not seem to ameliorate the toxicity. It is not seen with non-expressing vectors (61). Accordingly, the toxicity seems related to recombinant protein expression. Fortunately, the toxicity is mild and short lived, and importantly, does not seem to cause obvious symptoms in the non-human primates. DRG toxicity has been reported in human trials of rAAV, but not for hemophilia (1, 2, 54, 62). However, it does not appear that subjects enrolled in hemophilia rAAV gene therapy trials were carefully evaluated for DRG toxicity, or if they were, this data is not reported in published manuscripts (1, 2, 24, 25).

## Conclusion

The past 2–3 decades have seen tremendous advances in the treatment of hemophilia. Unlike many other genetic diseases, hemophilia patients can expect a near normal lifespan with controllable morbidities. The burden of treatment remains but is becoming lighter. Thus, any new treatments are tasked with demonstrating superior efficacy (reduced bleeding risk, medication administration, morbidity, mortality, costs, etc.) to current and near future treatments with little to no added risk. In other words, gene therapy must have “flawlessly safety” (63). Gene therapy for hemophilia using rAAV does not appear to meet those requirements.

Long term data with rAAV gene therapy is lacking. Although indirect evidence points toward the possibility of normalizing the lifespan of some hemophilia patients, that remains to be proven. Hemophilia gene therapy may reduce the risk of hemophilic arthropathy that develops during adulthood. Because rAAV gene therapy will not be given to children, it will have no impact on joint disease that develops prior to adulthood. The lifetime risk of joint disease in people in the general population is well over 50% (64). Thus, even if it is shown that gene therapy reduces the risk of hemophilic arthropathy, large numbers of hemophilia patients will still develop joint disease. The inconvenience of monthly subcutaneous injections currently or soon to be available for hemophilia remains. However, in comparison to most chronic diseases in which daily administration of medication is needed, the burden of hemophilia treatment is no longer onerous. Hence, the benefits of hemophilia gene therapy with rAAV are marginal.

It is often claimed that the risk of hemophilia gene therapy with rAAV is “low,” but what exactly is meant by “low” is not defined (2, 35, 62). If the latency for development of HCC with rAAV is like that of hepatitis B and C, we will not know the true risk for HCC following rAAV gene therapy for several decades. Clinical trials are not powered to detect rare complications. Therefore, the risk of hemophilia gene therapy with rAAV cannot currently be quantified. It is not zero and is potentially catastrophic. In addition, a proper epidemiological investigation of the growing number of reports of cancer following rAAV gene therapy for hemophilia is needed. Also needed are investigations of DRG toxicities in hemophilia patients following rAAV gene therapy. The reason(s) for falling factor VIII activity following rAAV gene therapy remain unresolved and need clarification before rAAV is widely used for this disorder.

Gene therapy for hemophilia was first proposed when the complications of this disorder were substantial. In the decades it has taken for rAAV gene therapy to become successful, gains in the standard of care have become revolutionary. Any new treatment that is “low” risk may be too high of a risk for hemophilia patients. All new treatment requires an assessment of risks and benefits. Despite the rare fatal risks due to rAAV for SMA, the benefits still outweigh the risks for this disorder. Based on the data presented here, that does not seem to be the case for hemophilia gene therapy with rAAV.

## Data availability statement

The raw data supporting the conclusions of this article will be made available by the author, without undue reservation.

## Author contributions

JP: Writing – original draft, Conceptualization, Investigation.
